# Land–atmosphere feedbacks contribute to crop failure in global rainfed breadbaskets

**DOI:** 10.1038/s41612-023-00375-6

**Published:** 2023-05-29

**Authors:** Hao Li, Jessica Keune, Femke Smessaert, Raquel Nieto, Luis Gimeno, Diego G. Miralles

**Affiliations:** 1 Hydro-Climate Extremes Lab, Ghent University, Ghent, Belgium; 2 Environmental Physics Laboratory (EPhysLab), Universidade de Vigo, Ourense, Spain

**Keywords:** Climate-change mitigation, Atmospheric dynamics, Agroecology

## Abstract

Global crop yields are highly dependent on climate variability, with the largest agricultural failures frequently occurring during extremely dry and hot years. Land–atmosphere feedbacks are thought to play a crucial role in agricultural productivity during such events: precipitation deficits cause soil desiccation, which reduces evaporation and enhances sensible heating from the land surface; the amplified local temperatures and moisture deficits can be detrimental to crop yield. While this impact of local land–atmosphere feedbacks on agricultural productivity has recently been reported, the dependency of crop yields on upwind regions remains understudied. Here, we determine the spatio-temporal origins of moisture and heat over the world’s largest 75 rainfed breadbaskets, and illustrate the crop yield dependency on upwind regions. Further, we disentangle the role of local and upwind land–atmosphere interactions on anomalous moisture and heat transport during low-yield years. Our results indicate that crop failure increases on average by around 40% when both upwind and local land–atmosphere feedbacks cause anomalously low moisture and high heat transport into the breadbaskets. The impact of upwind land–atmosphere feedbacks on productivity deficits is the largest in water-limited regions, which show an increased dependency on moisture supply from upwind land areas. Better understanding these upwind–downwind dependencies in agricultural regions can help develop adaptation strategies to prevent food shortage in a changing climate.

## Introduction

Ensuring food security for an increasing population has become one of the biggest human challenges in recent decades^1^. Dry and hot spells may cause crop failure^2–7^, thus their potential aggravation due to global warming increases the magnitude of the challenge^8^. That crop growth is sensitive to climate variations is known since the origins of agricultural settlements^9^. Today, approximately one-third of the global inter-annual crop yield variability is attributed to fluctuations in weather and climate^10^, albeit the relative importance of local climate variability remains in dispute^11^. While irrigation can mitigate water and heat stress on crops^12–14^, it also poses severe pressure on global freshwater resources^15^. Therefore, despite its high dependency on precipitation and temperature^16^, rainfed agriculture stands as a sustainable means to meet food demands while preventing the overexploitation of surface and groundwater resources. Currently, ~75% of global croplands and ~60% of global food production is owed to rainfed agriculture^17,18^. In addition, a great share of the world’s smallholder farmers relies on rainfed agriculture for their livelihoods^19,20^, emphasizing its importance for stabilizing local socioeconomic systems. Consequently, understanding the climate vulnerability of global rainfed ’breadbaskets’ is essential to prioritize adaptation strategies that strive towards securing local food supply in light of future changes in climate.

Heat stress may not only directly damage plant tissues^21^, but also contribute to soil moisture deficit and atmospheric aridity while increasing autotrophic respiration^22^. Local land–atmosphere feedbacks have been hypothesized to play a crucial role in this process: as soils dry out, temperatures and atmospheric aridity are further enhanced^23^, which may exacerbate water deficits and heat stress and reduce crop yields^24,25^. These local land–atmosphere feedbacks are conditioned on the occurrence of specific large-scale synoptic systems^26^. Climate oscillations, such as the El Niño Southern Oscillation (ENSO) and the North Atlantic Oscillation (NAO), are known to influence local climate and land–atmosphere feedbacks, and have been reported to instigate crop loss^27,28^, sometimes in a worldwide synchronous fashion^29,30^. For example, a recent study further emphasizes that certain Rossby wave configurations can increase the exposure of agricultural regions to heatwaves and induce simultaneous crop failure across many regions in the Northern Hemisphere^31^. These large-scale atmospheric patterns may induce preferential synoptic conditions and thus affect the entire flow of heat and moisture in the atmosphere. The subsequent anomalies in the imports of heat and moisture to breadbasket regions may translate into local temperature and precipitation anomalies. These anomalies can then be enhanced or attenuated by the above-mentioned local land–atmosphere feedbacks^26^. Moreover, land–atmosphere feedbacks can further lead to the spatial propagation of dry and hot anomalies, as their local influence further translates into anomalies of heat and moisture advection downwind^32–34^, and may even affect larger-scale synoptic patterns^35–37^. The larger-scale influence of land–atmosphere feedbacks on downwind advection and atmospheric circulation could then contribute to a downwind propagation of adverse weather conditions for crops, within and across breadbaskets.

Although a few recent studies have analysed the influence of local land–atmosphere feedbacks on crops^24,25^, little is known about the dependency of agricultural yields on upwind climate conditions. Here, we first examine 75 global rainfed breadbaskets and analyse their crop yield variability, focusing on four major food crops: maize, wheat, soybean, and rice. Low-yield years are studied in relation to local climate anomalies. Then, using a novel atmospheric Lagrangian modelling framework, along with satellite observations (see Methods), we investigate the sources of heat and moisture during these low-yield years, the local and upwind contributions to the atmospheric heat budget and precipitation, and the potential influence of land–atmosphere feedbacks on those processes. A better understanding of the role of upwind land regions may improve seasonal crop forecasting, facilitate the development of early-warning systems in global breadbaskets, and help target adaptation strategies that aim to minimize the risk of large-scale crop failure.

## Results

### Crop failure events and local climate anomalies

**Fig. 1:**
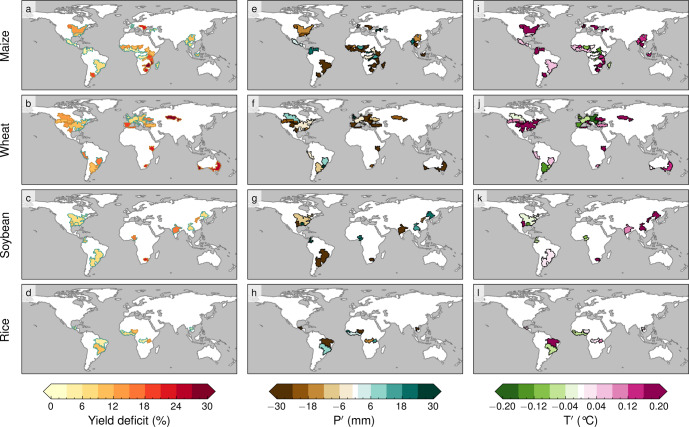
Yield deficits and local climate anomalies in global breadbaskets during crop failure events. **a**–**d** Average yield deficits (%) during crop failure events for 75 rainfed breadbaskets, including **a** maize, **b** wheat, **c** soybean and **d** rice. Green contours show energy-limited (EL; *E*_*p*_/*P* < 1.0) breadbaskets, and brown contours indicate water-limited (WL; *E*_*p*_/*P* > 1.0) breadbaskets. **e**–**h** Average precipitation anomalies (${P}^{{\prime} }$, mm) during crop failure events. **i**–**l** Average 2-meter air temperature anomalies (${T}^{{\prime} }$, ^∘^C).

Here, we define crop failure events as the years when the relative yield anomaly, calculated by removing any long-term changes associated with technological improvements, falls below the 25th percentile at each specific rainfed breadbasket (see Methods). We consider the period 1983–2015, and a total of 75 global breadbaskets dedicated to maize, wheat, soybean and rice (Fig. 1a–d), resulting in a total of 675 crop failure events. The average deficit in crop yield during these events shows considerable spatial variation among breadbaskets (Fig. 1a–d), with wheat exhibiting the largest average loss (~15%) and rice the lowest (~7%). For individual breadbaskets, severe productivity deficits of up to 50% are found in certain years. Moreover, water-limited breadbaskets generally show higher losses than energy-limited ones (*p* value < 0.05, Supplementary Fig. 1a–d). This stems from the large inter-annual climate variability in water-limited regions and the importance of moisture availability for rainfed crops (see Supplementary Fig. 1e–h). Examples of water-limited regions that suffer larger yield deficits include Eastern and Southern Africa for maize, the Iberian Peninsula, Eastern Europe, Eastern America and Australia for wheat, and India for soybean (Fig. 1a–d).

*P* deficits (i.e. brown colours in Fig. 1e–h) and high *T* (i.e. red colours in Fig. 1i–l) are frequently associated with crop failure, which is indicative of dry and hot growing seasons having an overall detrimental influence on yield. This is particularly the case in water-limited breadbaskets; most extra-tropical breadbaskets experience high T and low P independently of the crop, with few exceptions—such as the Iberian Peninsula, Eastern Europe and the Pampas region—where wheat losses tend to be associated with dry and cold (instead of hot) growing seasons (Fig. 1f, j). In addition, the highly positive correlations between yield anomalies and aridity index (i.e., potential evaporation (*E*_*p*_) over precipitation, *E*_*p*_/*P*) also support the high sensitivity to *P* in water-limited breadbaskets (Supplementary Fig. 2). In energy-limited regions, and particularly over the tropics, the dependency of yield on *P* and *T* is more complex, and some areas—such as maize breadbaskets in Venezuela and Colombia and some regions in Africa—experience higher-than-usual *P* during low-yield years (green colours in Fig. 1e–h). Higher-than-usual P may cause waterlogging^25^ and further come along with decreased radiation^38^, with both factors being detrimental to crop growth. Due to the association of crop failure with anomalously dry and hot years in most global breadbaskets, particularly in water-limited ones, land–atmosphere feedbacks are expected to play an aggravating role.

### Sources of atmospheric moisture and heat

**Fig. 2:**
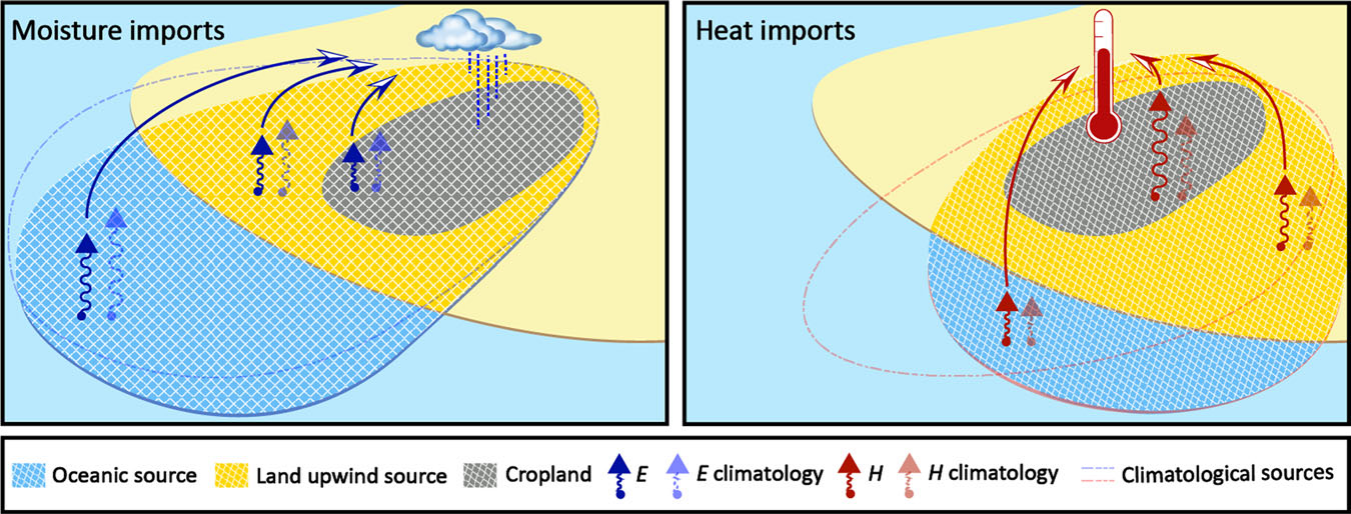
Source regions of moisture and heat during crop failure events. Source regions of moisture and heat (blue and red contours) delineate the areas from which the air over the breadbasket was previously moistened (through evaporation, *E*, blue arrows) and warmed (through surface sensible heat flux, *H*, red arrows). Source region contributions can be subdivided into the contributions from ocean and surface waters (blue), upwind land (yellow), and local land within the breadbasket (grey). The sum of all contributions is the total import of heat and moisture into the atmosphere over the breadbasket (see Methods). The relative import contributions are bias-corrected with reanalysis and satellite data (see Methods). During crop failure events, the source regions may shift from the climatological ones (compare solid blue and dashed blue lines, and solid red and dashed red lines), and land--atmosphere feedbacks may influence the contribution from upwind and local land (compare light blue and dark blue arrows, and light red and dark red arrows). In particular, dry and hot conditions may trigger land--atmosphere feedbacks that cause a decreasing moisture import but an increasing heat import from upwind and local land.

To reveal the origins of the atmospheric moisture and heat imports into the biggest 75 rainfed breadbaskets, we perform a backward tracking of heat and moisture from each breadbasket region^39^—see Methods. Simulations with 2 million air parcels globally are used, and those parcels residing over the breadbaskets at certain time steps are traced back in time for up to 15 days. This enables us to identify the upwind regions where the air was warmed and moistened through evaporation and surface sensible heat, respectively. We track the air over each breadbasket for all growing seasons between 1983–2015, and disentangle the moisture and heat contributions from (i) oceans and surface waters, (ii) upwind land, and (iii) local land (i.e., within the breadbasket). The time series of these contributions are used to evaluate and delineate the climatological moisture and heat source regions, as well as their anomalies during crop failure events (Fig. 2). During those low-yield years, dry soils (locally, but also upwind) may trigger land–atmosphere feedbacks that cause decreasing moisture imports and increasing heat imports. A conceptual diagram illustrating these moisture and heat sources and their potential anomalies during crop failure events is shown in Fig. 2.

**Fig. 3:**
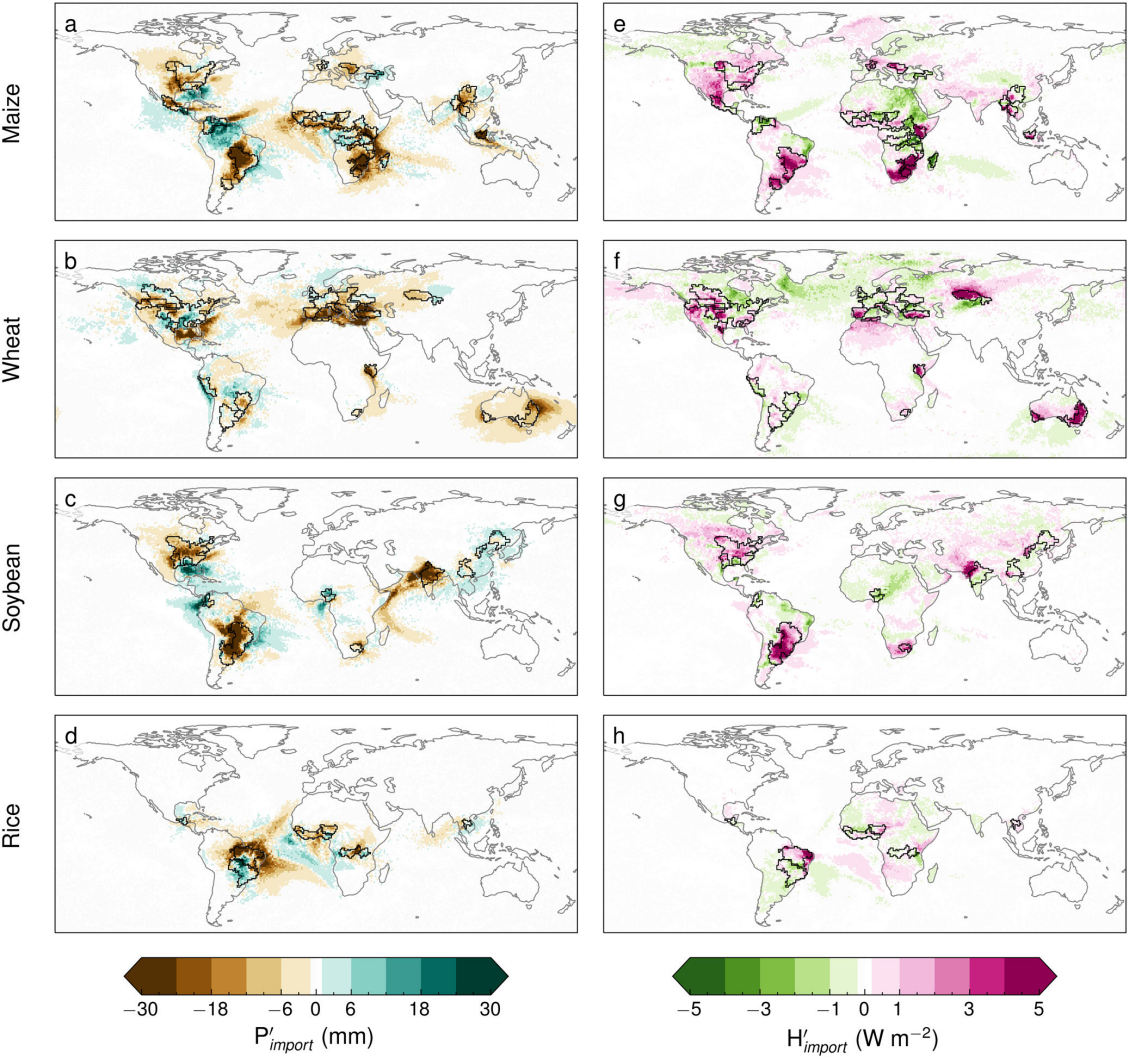
Anomalies in moisture and heat imports to global rainfed breadbaskets during crop failure events. Anomalies in (**a**–**d**) moisture leading to precipitation (${P}_{import}^{{\prime} }$, mm) and in (**e**–**h**) heat (${H}_{import}^{{\prime} }$, W m^−2^) imported into the breadbaskets. Results are illustrated for all four crop types: maize (**a**, **e**), wheat (**b**, **f**), soybean (**c**, **g**) and rice (**d**, **h**). Anomalies are averaged over all crop failure events and summed over all breadbaskets when overlapping (see Methods). Source region anomalies for individual breadbaskets are shown in ref. ^54^.

Average moisture and heat sources reveal the importance of upwind ocean and land regions for the climate conditions over the world’s major breadbaskets (Supplementary Fig. 3). During crop failure events, moisture and heat imports are often anomalous, with these anomalies extending far upwind from the agricultural regions (Fig. 3). For moisture, nearby oceans typically provide less moisture for precipitation than usual during these events (brown-coloured regions in Fig. 3), although some of these ocean regions also experience positive anomalies, indicating their increased contribution to precipitation over the respective breadbaskets during low-yield years (green-coloured regions in Fig. 3). These positive anomalies occur more frequently for breadbaskets located in tropical energy-limited regions and may be explained by the lower availability of incoming solar radiation or waterlogging in periods of high moisture advection and precipitation, as discussed above. The strongest negative anomalies in moisture and positive heat contributions during crop failure events occur, however, within the breadbaskets themselves, and over nearby land regions (see darker brown and red colours in Fig. 3). This is particularly the case for heat, in which anomalies almost always originate over land, within the breadbaskets and their close surroundings. For most breadbaskets, and particularly for the water-limited ones, the signal is clear: lower-than-usual moisture and higher-than-usual sensible heat contributions are found during crop failure events, and often these anomalies (relative to the climatology) are larger closer to the breadbaskets (see brown-coloured regions in Fig. 3a–d and red-coloured regions in Fig. 3e–h). A noticeable exception is the unusually high moisture contribution from Amazonia to the maize breadbaskets in Venezuela and Colombia during crop failure events (see green colours in Fig. 3a).

### Land–atmosphere feedbacks during crop failure events

During crop failure events, often the air progressively dries and warms as it travels from the ocean towards the breadbaskets. Figure 3 illustrates how negative oceanic moisture import anomalies tend to intensify as soon as the air reaches the continents, and concurrent positive heat anomalies concur in the air’s journey towards the breadbasket from the upwind land regions (see progressively darker brown and red colours in Fig. 3). Prominent examples of the occurrence of these ’en route feedbacks’ are Eastern and Southern Africa for maize breadbaskets (Fig. 3a, e), North America and Eastern Australia for wheat (Fig. 3b, f), and India, North America and the Pampas region for soybean (Fig. 3c, g). The intensification of land–atmosphere coupling as the air travels from ocean to land is expected, since low moisture transport from the ocean can lead to reduced precipitation inland, induce land dry-out and hence reinforce positive soil moisture–temperature and –precipitation feedbacks^23^. In that sense, the land tends to amplify the anomalies in heat and moisture imports from the ocean, which originally result from synoptic-scale climate patterns^26^. These positive feedbacks act en route from the upwind land regions to the breadbasket and potentially intensify along the way—see also Fig. 2.

**Fig. 4:**
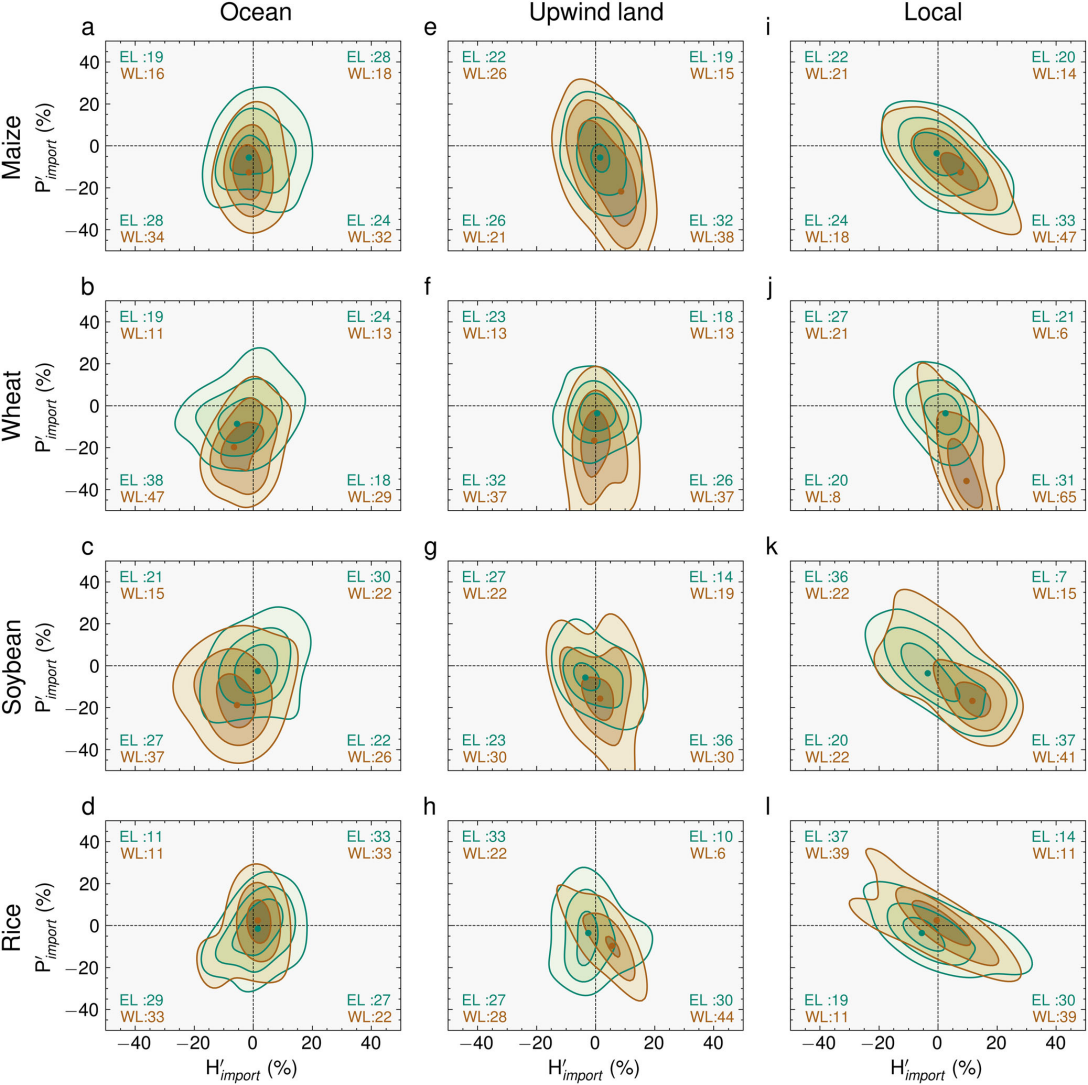
Yield deficits as a function of heat and moisture import anomalies from ocean, upwind land and local land. Each column describes one possible origin: ocean (**a**–**d**), upwind land (**e**–**h**) and local (**i**–**l**). Each row illustrates one of the four crop types: maize (**a**, **e**, **i**), wheat (**b**, **f**, **j**), soybean (**c**, **g**, **k**) and rice (**d**, **h**, **l**). Heat import anomalies (${H}_{import}^{{\prime} }$, %) from each origin are shown on the *x*-axis and moisture import anomalies (${P}_{import}^{{\prime} }$, %) on the *y*-axis of each plot. Contours illustrate two-dimensional probability densities and the points indicate the centre of gravity of the distributions, as estimated with crop-loss-weighted Gaussian kernels^55^. Green points and contours are based on all events from energy-limited breadbaskets (EL; *E*_*p*_/*P* < 1.0); brown points and contours are based on all events from water-limited breadbaskets (WL; *E*_*p*_/*P* > 1.0). Each subplot is further subdivided into four quadrants indicating either positive or negative moisture and heat import anomalies. The brown and green numbers in each quadrant refer to the percentage of all events from water- and energy-limited breadbaskets located in the corresponding quadrant. Note that axes are cropped to [−50,50] (*x*-axis) and [−50,50] (*y*-axis) for visualization purposes.

Therefore, anomalies over remote land regions with both negative moisture contributions and positive heat contributions suggest that upwind land–atmosphere feedbacks play a role in the heat and moisture imports (see also blue and red arrows in Fig. 2). To further unravel the relative importance of these different contributions during crop failure events, we subdivide the origins of heat and moisture into ’ocean’, ’upwind land’ and ’local land’, and map the yield anomaly as a function of the heat and moisture imports from those three for all crop failure events over water- and energy-limited breadbaskets (brown and green points and contours, respectively, Fig. 4). Points mark the centre of gravity, while the surrounding contour lines represent probability densities. The proportion of crop failure events in each quadrant is indicated by the percentages in each corner (see also Supplementary Fig. 4, again separately for energy-limited (green) and water-limited (brown) breadbaskets). Altogether, the distributions in Fig. 4 illustrate the dependency between heat and moisture imports and yield loss.

Crop failure events are typically associated with precipitation deficits that originate in the ocean; this is particularly clear for maize, wheat and soybean, where the majority of events fall into one of the lower two quadrants associated with moisture deficits (Fig. 4a–c). This pattern is significant for water-limited breadbaskets (*p* value < 0.05, Supplementary Fig. 4e–h), all crop types in water-limited regions experience below-normal moisture imports from the ocean in ~60% of all events (Fig. 4a–d). The strongest ocean signal is found for water-limited wheat breadbaskets, which experience below-normal moisture imports from the ocean in 76% of crop failure events (Fig. 4b). Exceptions are the energy-limited maize and soybean breadbaskets, where no anomalies in oceanic moisture and heat imports are evident during crop failure events (*p* value > 0.05, Supplementary Fig. 4a, c).

After the air parcels reach the continents en route to the breadbasket, they tend to gain less moisture during crop failure events than they usually do. This is evidenced by the fact that the majority of events fall into the lower two quadrants in Fig. 4e–h, and are thus associated with moisture deficits from upwind land regions. In contrast to the ocean contributions, we find that the upwind land tends to warm the air parcels disproportionately during these low-yield years—i.e., more events fall into the lower right quadrant in Fig. 4e–h. This ’upwind feedback’ is also stronger for water-limited breadbaskets, especially for maize and wheat (*p* value < 0.05, Supplementary Fig. 4e, f); 38% and 37% of crop failure events are associated with both negative moisture and positive heat anomalies from upwind land (Fig. 4e, f). In energy-limited regions, on the other hand, the higher density in the centre of the plot (green points and contours in Fig. 4e–h) indicates a lower dependence of crop failure events on upwind land feedbacks in these breadbaskets (*p* value > 0.05, see also Supplementary Fig. 4a, b).

Finally, when the already anomalously warm and dry air parcels arrive over the breadbasket, local land–atmosphere feedbacks further aggravate these dry and hot anomalies (Fig. 4i–l). This is suggested by the additional increase in heat anomalies and decrease in moisture anomalies within the breadbaskets themselves, illustrated by a shift of the density contours towards larger heat and moisture anomalies in the lower-right quadrant in Fig. 4i–l. For example, for the water-limited breadbaskets dedicated to growing maize and wheat, 47% and 65% of crop failure events (respectively) experience both anomalous drying and warming of the overlaying atmosphere from the land surface during crop failure events (*p* value < 0.05, Supplementary Fig. 4e, f). Nonetheless, a considerable fraction of crop failure events occur during wetter and colder conditions than usual, especially in energy-limited regions (see the upper-left quadrant in Fig. 4i–l).

### Concurrent land–atmosphere feedbacks amplify yield deficits

**Fig. 5:**
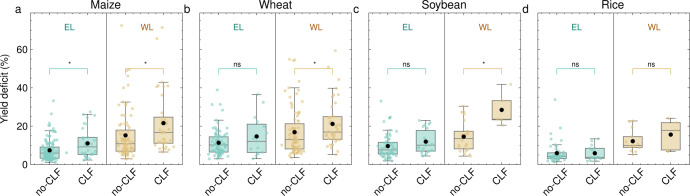
Yield deficits and their relation to local and upwind land–atmosphere feedbacks. Crop failure events per crop type (maize (**a**), wheat (**b**), soybean (**c**), rice (**d**)), and for energy-limited (green) and water-limited (brown) breadbaskets. Events are grouped into two categories: those associated with concurrent local and upwind land–atmosphere feedbacks ('CLF')—i.e., positive anomalies in heat imports and negative anomalies in moisture imports in both local and upwind land—and all others ('no-CLF'). The box spans from the lower to the upper quartile, encompassing the interquartile range (IQR, Q3–Q1). The lower whisker extends to the first data point greater than Q1 − 1.5*IQR; the upper whisker extends to the last data point smaller than Q3 + 1.5*IQR. Black dots indicate the mean anomaly in yield, and coloured dots indicate the yield anomaly for individual events. The differences between 'no-CLF' and 'CLF' are assessed by the Mann–Whitney *U* test^51^, in which '*' indicates significant differences (*p* value < 0.05), and 'ns' indicates non-significant differences (*p* value > 0.05).

As indicated above, the occurrence of yield deficits is dependent on local but also remote land–atmosphere feedbacks: crop failure events are often associated with lower-than-usual moisture imports and higher-than-usual heat imports, with increasing anomalies from upwind to local land regions (Fig. 4). Here, we evaluate the potential influence of concurrent, positive local and upwind land–atmosphere feedbacks on crop yield. To do so, Fig. 5 shows the yield deficit for all four crops separated into the water- and energy-limited regions and grouped into two categories: (i) yield anomalies associated with positive heat anomalies and negative moisture anomalies from both upwind and local land regions (denoting concurrent local and upwind land–atmosphere feedbacks, CLF), and (ii) all other events (no-CLF). Note that the latter also includes, for instance, crop failure events for which only local positive land–atmosphere feedbacks take place, or events for which upwind and local contributions are anomalously negative for moisture but not for heat. Despite this rather rigorous categorization, we find that, for all crops, the yield deficit is higher when anomalous upwind and local land conditions combine, contributing to the dry-out and warm-up of the atmosphere over the breadbaskets (compare black points in Fig. 5a–d). As expected, larger differences are found for water-limited breadbaskets (*p* value < 0.05, brown boxplots in Fig. 5a–c), except for the case of rice (Fig. 5d). Nonetheless, the magnitude of crop failure events in energy-limited breadbaskets is also disproportionately larger when local and upwind land–atmosphere feedbacks concur, especially for maize (*p* value < 0.05) (see green boxplots in Fig. 5a–d).

While the largest mean difference is observed for water-limited soybean breadbaskets, it is noted here that only one of those breadbaskets experienced concurrent local and upwind land–atmosphere feedbacks (see brown points in Fig. 5c), and that there are generally few events in rainfed soybean breadbaskets. The same is true for rice (Fig. 5d). For maize and wheat, enough breadbaskets are located in both energy- and water-limited regions, and many crop failure events are associated with positive land–atmosphere feedbacks throughout the entire air trajectory over land (37 out of 117 cases for maize, 41 out of 126 cases for wheat). In water-limited regions, when upwind and local land regions contribute to anomalously low precipitation along with increased sensible heating, the average deficit in yield is 37% higher. For maize and wheat, significant differences are seen over water-limited breadbaskets between no-CLF and CLF (*p* value < 0.05): 43% higher (from 15.2 to 21.6%) for maize, and 25% higher (from 16.9 to 21.2%) for wheat. In energy-limited regions, the average deficit in yield is 48% higher (from 7.4 to 11.1%, *p* value < 0.05) for maize, and 29% (from 11.4 to 14.7%, *p* value > 0.05) for wheat. Averaged over all breadbaskets and events, crop failures are 42% larger when both upwind and local land–atmosphere feedbacks cause anomalously low moisture transport along with anomalously high heat transport into the breadbaskets.

## Discussion

The influence of local land–atmosphere feedbacks on agricultural productivity has long been assessed, yet the origins of the climate anomalies triggering these feedbacks remained understudied. Here, we analysed the 75 largest rainfed breadbaskets worldwide to better understand the dependencies of yield on local and upwind land surface conditions. Using a Lagrangian modelling framework, we delineated the source regions of moisture and heat for these agricultural lands. Focusing on crop failure events over each of those breadbaskets, our results suggest that not only local and upwind land–atmosphere feedbacks tend to amplify local climate anomalies, but that the severity of crop failure events is statistically (and significantly) dependent on the magnitude of these feedbacks. The highest dependency is observed for wheat and maize breadbaskets in water-limited regions. There, the average yield deficit for all crops increases by 42% when both upwind land and local land provide less moisture and more heat than usual, which is indicative of a strengthening of land–atmosphere coupling triggered by reduced precipitation in the upwind land sources.

Our study focuses on the climatic drivers of year-to-year fluctuations in yield, and in particular on the influence of precipitation and temperature dynamics. As such, the impacts of inter-annual technological changes or land use management on crop yield are not directly accounted for. It is assumed that incorporating these dynamics results in a higher fraction of unexplained variance in yield variability. Moreover, the extension of the global breadbaskets is considered static in time. As such, we do not consider the expansion of agricultural area or the conversion of rainfed to irrigated croplands, nor any potential poleward migration of rainfed agriculture due to global warming^40^. Nonetheless, our results suggest an important role of upwind land management for the productivity of agricultural lands, due to the impact that land management has on the climate and thus on the imports of moisture and heat that modulate precipitation and temperature in the breadbaskets. For example, deforestation upwind may decrease moisture recycling and cause reduced moisture advection and precipitation in the breadbaskets, potentially increasing crop failure risks^41^. Moreover, an important implication of the demonstrated dependency on upwind land conditions relates to the forecasting of crop failure events. Given the memory of soil moisture and vegetation conditions, satellite monitoring of upwind land sources can help improve the accuracy of seasonal crop forecasts and help provide data for those regions with a scarcity of local observations. Recent studies have already demonstrated that upwind extreme events, such as droughts and heatwaves, are likely to propagate downwind and be fuelled by the decrease in evaporation and enhanced sensible heat from the land surface^32,33^. Improved mechanistic understanding of these processes can help forecast climate anomalies in downwind breadbaskets, especially for those water-limited where crop yield is highly dependent on advected moisture and heat, as shown here.

It is important to note that our results are conditioned on the uncertainties inherent to the evaluation of Lagrangian trajectories. For instance, the applied framework tends to yield moisture recycling ratios that are on the upper end of model-internal uncertainties^39^. Moreover, our use of Lagrangian trajectories can help reveal causal links between evaporation and sensible heat fluxes upwind, and precipitation and temperature downwind, yet the statistical relationships found between climate anomalies and yield are not necessarily causal, even if correlations agree with the physical interpretation of the Lagrangian trajectories constrained by observations. Needless to say that the sparsity of ground observations is a challenge in validating and bias-correcting the sources of precipitation, and particularly evaporation and sensible heat flux^39^. An option to verify the source contributions for moisture in the future is to monitor the isotopic composition of precipitation, but such observations remain scarce and often insufficient to understand the origins of rainfall^42^.

Overall, our results highlight the importance of upwind land management for the productivity of agricultural lands, and they may help improve seasonal crop forecasting and facilitate earlier and better management decisions. Further understanding the dependency of crop productivity on upwind climate may ultimately help develop robust adaptation strategies to secure food supply to our growing population in a changing climate.

## Methods

### Definition of breadbaskets

Here, we identified rainfed breadbaskets for four major crops: maize, wheat, soybean and rice. An agricultural region is considered to be a rainfed breadbasket if (i) the region encompasses at least 18 spatially connected grid cells on a global 1^∘^ grid, which corresponds to at least ~150.000 km^2^, (ii) the rainfed fraction in each of the pixels exceeds 75%, and (iii) all pixels within the region have the same sowing and harvest months. The rainfed area was determined using the global monthly irrigated and rainfed areas in the year 2000 (MIRCA2000^17^), and the rainfed fraction was calculated using the total agricultural area (i.e., the sum of rainfed and irrigated area) in each 1^∘ ^× 1^∘^ grid cell. These constraints result in a total of 75 rainfed breadbaskets (29 for maize, 25 for wheat, 12 for soybean and 9 for rice). To determine the main growing seasons, we used the crop calendar from ref. ^43^, which indicates the corresponding sowing and harvest months for each crop in the year 2000 (Supplementary Fig. 6). Yield data was retrieved from the global gridded crop yield dataset (GDHY^44^), which is available for 1981–2016 and constrains the period considered in this study. For each breadbasket *i* and each year *t*, the yields were aggregated using an area-weighted sum of the rainfed production:

1 $${Y}_{i,t}=\frac{\sum ({Y}_{i,t,g}\times {A}_{g})}{\sum {A}_{g}},$$

where *Y*_*g*_ is the yield of each *g*th 1^∘ ^× 1^∘^ grid cell in the breadbasket and *A*_*g*_ is the corresponding rainfed area (at least 75% of the total agricultural area in the grid cell *g*). The production of these major breadbaskets comprises ~74%, ~94% and ~13% of the global rainfed production for maize, soybean, and rice, respectively (Supplementary Fig. 5a, c, d). For wheat, both spring and winter seasons are considered; which, together, comprise ~80% of the global wheat production (Supplementary Fig. 5b). To remove any long-term trends associated with technological improvements, we detrended the crop yield using locally weighted scatter plot smoothing (LOWESS^29,45^) and get relative yield anomalies (*η*, %) for each breadbasket *i*:

2 $${\eta }_{i,t}=\frac{{Y}_{i,t}-{\mu }_{i,t}}{{\mu }_{i,t}}\times 100,$$

where *Y*_*i*,*t*_ is the actual yield and *μ*_*i*,*t*_ is the expected yield estimated by LOWESS for the year *t* over the breadbasket *i*.

Using the detrended time series, low-yield years are identified based on the relative yield anomalies for each breadbasket. Here, crop failure events are defined as the years in which yield was below the 25th percentile of all years between 1983–2015 (i.e., nine years), with that threshold aiming to secure a sufficient number of events for a robust statistical analysis. The average yield deficit during those years is calculated by averaging the relative yield anomalies for each breadbasket (Fig. 1a–d).

### Local climate anomalies

For the climatological assessment in Fig. 1e–l, climatological variables, such as 2-m temperature (*T*) and precipitation (*P*), were considered. To account for, e.g., the memory effects of soil moisture that significantly influence crop growth, we considered an extended growing season for these variables, i.e., the full growing season and one full month before the identified sowing month. The following data sets were used: precipitation from the Multi-Source Weighted Ensemble Precipitation (MSWEP^46^) v2.2, temperature from the European Centre for Medium-Range Weather Forecasts reanalysis (ERA-5^47^), evaporation and potential evaporation over land from the Global Land Evaporation Amsterdam Model (GLEAM^48^) v3.5a and evaporation over the oceans from the Objectively Analyzed air-sea Fluxes for the global oceans dataset (OAFlux^49^). Climate anomalies during low-yield years were also averaged over all nine crop failure events (e.g., in Fig. 1e–l). To differentiate between water- and energy-limited breadbaskets, the aridity index was employed. We calculated the aridity index using the climatological annual average of *E*_*p*_/*P*, with *E*_*p*_ being potential evaporation, between 1983–2015 (see contours in Fig. 1a–d). Water-limited breadbaskets were defined where *Ep*/*P* > 1 and energy-limited breadbaskets were defined where *Ep*/*P* < 1^50^. In addition, we use the Mann–Whitney *U* test^51^ to assess the significance of differences in climatic variables between water- and energy-limited regions.

### Heat and moisture tracking framework

To determine the spatio-temporal origins of heat and moisture over each identified breadbasket, a novel Heat And MoiSture Tracking framEwoRk (HAMSTER^39^) was employed. The framework is based on the Lagrangian particle dispersion model FLEXPART v9.01^52^ and tracks air parcels in space and time. Here, FLEXPART was driven with ERA-Interim reanalysis^53^ at 1^∘^ resolution and at three-hourly time steps; the analysis was performed on six-hourly reanalysis time steps. Simulations were performed globally, with two million air parcels of equal mass distributed homogeneously. Each of these air parcels was then tracked for 40 years (1979–2019); however, analysis was constrained to 1983–2015 due to the availability of yield data (see above). For each of those two million air parcels, the following variables were tracked: longitude, latitude, height, specific humidity content, density, and (potential) temperatures; enabling the calculation of, e.g., the absolute humidity content of each parcel. The trajectories of air parcels residing over each breadbasket were then evaluated to determine the source regions of heat and moisture for each breadbasket, i.e., the regions in which air parcels arriving over each breadbasket were moistened and warmed by evaporation and sensible heating from the surface. Therefore, air parcels residing over the breadbaskets each day between one month prior to the sowing month and the harvest month were identified and traced back in time for 15 days. The resulting backward trajectories were evaluated to establish source-receptor relationships of heat and moisture, accounting for rain en route and nighttime cooling (for details, see ref. ^39^). The resulting source-receptor relationships were bias-corrected with precipitation from MSWEP^46^ and evaporation from GLEAM^48^ and OAFlux^49^, and with sensible heat from ERA-Interim^53^. The resulting source regions depict the contributions of surface evaporation to precipitation (here also referred to as moisture imports) and sensible heating from the surface (here also referred to as heat imports) to energy over each of the 75 breadbaskets.

To unravel the contribution of land–atmosphere feedbacks in the breadbasket and from upwind regions on yield, we further distinguish between heat and moisture imports that are ’local’ (i.e. the land area within each breadbasket, ${P}_{import}^{l}$ and ${H}_{import}^{l}$), from ’upwind land’ (${P}_{import}^{u}$ and ${H}_{import}^{u}$) and from ’ocean’ (${P}_{import}^{o}$ and ${H}_{import}^{o}$). For each breadbasket *i*, the budget of heat and moisture in the year *t* was defined as follows:

3 $$\begin{array}{l}{P}_{import}^{i,t}={P}_{import}^{i,t,l}+{P}_{import}^{i,t,u}+{P}_{import}^{i,t,o}\\ {H}_{import}^{i,t}={H}_{import}^{i,t,l}+{H}_{import}^{i,t,u}+{H}_{import}^{i,t,o}\\ \end{array}$$

where the superscripts *l*, *u* and *o* represent the heat or moisture that is contributed locally, from upwind land and from ocean, respectively. For example, the relative anomalies (%) of heat imports ${H}_{import}^{{\prime} ,i,o}$ and moisture ${P}_{import}^{{\prime} ,i,o}$ imports from the ocean (superscript *o*) over the breadbasket *i* were calculated as follows:

4 $$\begin{array}{l}{P}_{import}^{{\prime} ,i,o,t}=\frac{\left({P}_{import}^{i,o,t}-\,\overline{{P}_{import}^{i,o}}\right)}{\overline{{P}_{import}^{i,o}}}\times 100\\ {H}_{import}^{{\prime} ,i,o,t}=\frac{\left({H}_{import}^{i,o,t}-\,\overline{{H}_{import}^{i,o}}\right)}{\overline{{H}_{import}^{i,o}}}\times 100\end{array}$$

Figure 4 was created using these relative anomalies of heat and moisture imports and relative yield anomalies during crop failure events.

### Supplementary information

Supplementary

## Data Availability

ERA-Interim data were accessed from http://apps.ecmwf.int/datasets. GLEAM data are available through https://www.gleam.eu. OAFlux data can be retrieved from https://oaflux.whoi.edu/data-access. MSWEP data are available through http://www.gloh2o.org. The FLEXPART model can be downloaded via https://www.flexpart.eu. Spatio-temporal origins of moisture and heat over the world’s largest 75 rainfed breadbaskets^54^ can be accessed by 10.6084/m9.figshare.21948542.v2.

## Code availability

The framework for analysis of FLEXPART data^39^ is available via https://github.com/h-cel/hamster. Python scripts for the analysis are available upon request from the corresponding author.
