# Supplementary material for: Land–atmosphere feedbacks contribute to crop failure in global rainfed breadbaskets
Source: NPJ Clim Atmos Sci. 2023 May 29;6(1):51. doi: 10.1038/s41612-023-00375-6 (PMC11041704; doi:10.1038/s41612-023-00375-6)
Supplement: Supplementary file 1 — Supplementary [file 41612_2023_375_MOESM1_ESM.pdf]

Land–atmosphere feedbacks contribute to crop failure in global rainfed breadbaskets

Hao Li[1,*], Jessica Keune[1], Femke Smessaert[1], Raquel Nieto[2], Luis Gimeno[2], and Diego G. Miralles[1,*]

[1]Hydro-Climate Extremes Lab, Ghent University, Ghent, Belgium

[2]Centro de Investigación Mariña, Universidade de Vigo, Environmental Physics Laboratory (EPhysLab), Ourense, Spain

[*]Hao.Liwork@UGent.be, Diego.Miralles@UGent.be

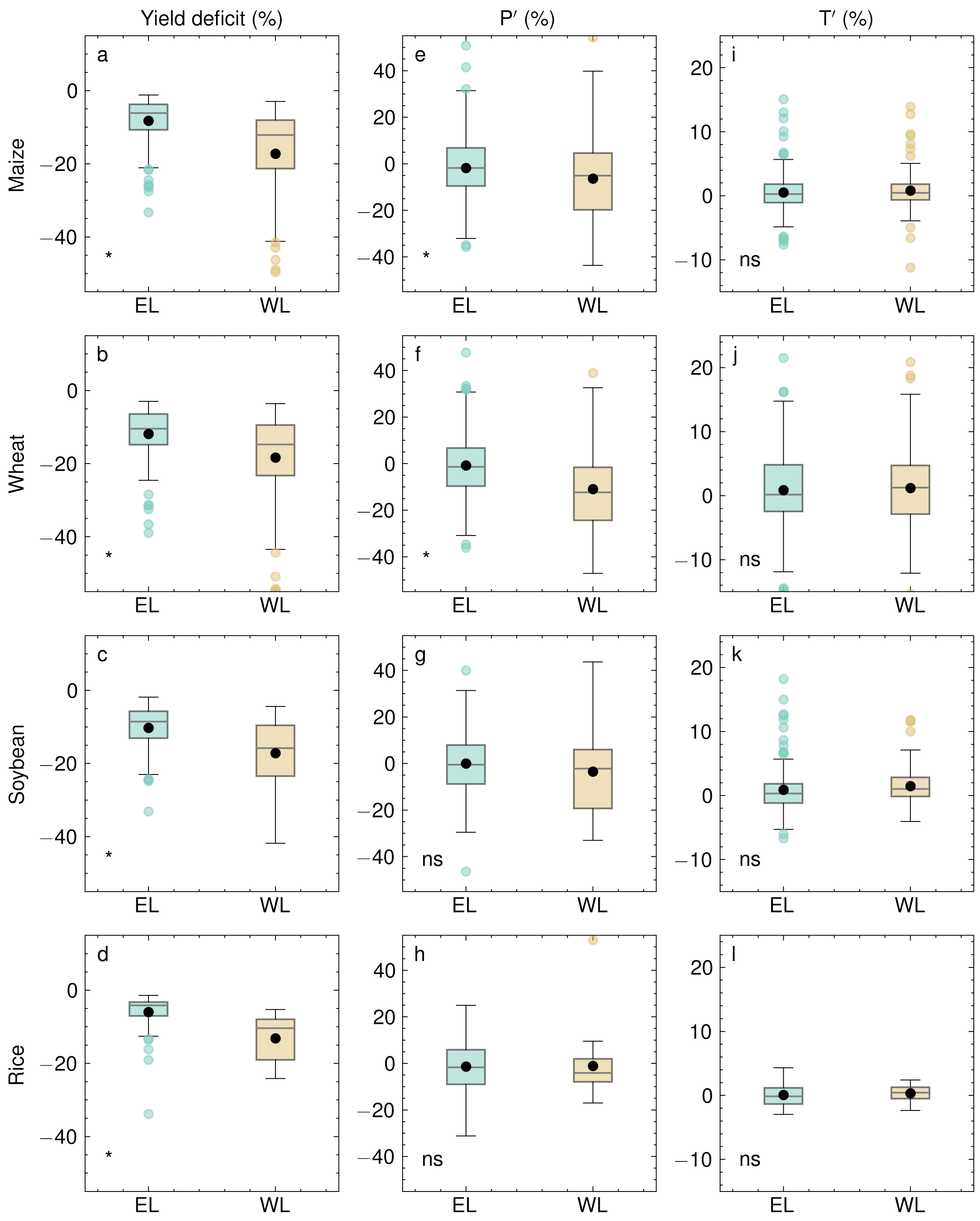

Supplementary Figure 1. Yield deficits and relative climate anomalies during crop failure events. **a–d** show yield deficits (%), **e–h** show average precipitation anomalies (P', %), and **i–l** show average 2-meter air temperature anomalies (T', %) during crop failure events. Boxes range from the lower to the upper quartile, encompassing the interquartile range (IQR, Q3–Q1). The lower whisker extends to the first data point greater than Q1 – 1.5*IQR; the upper whisker extends to the last data point smaller than Q3 + 1.5*IQR. Black dots indicate the mean anomaly, and coloured dots indicate outliers. Significant differences between energy-limited (EL; $E_p/P < 1.0$; green) and water-limited (WL; $E_p/P > 1.0$; brown) breadbaskets are assessed by Mann–Whitney U test — '*' indicates p value smaller than 0.05, and 'ns' indicate non-significance differences.

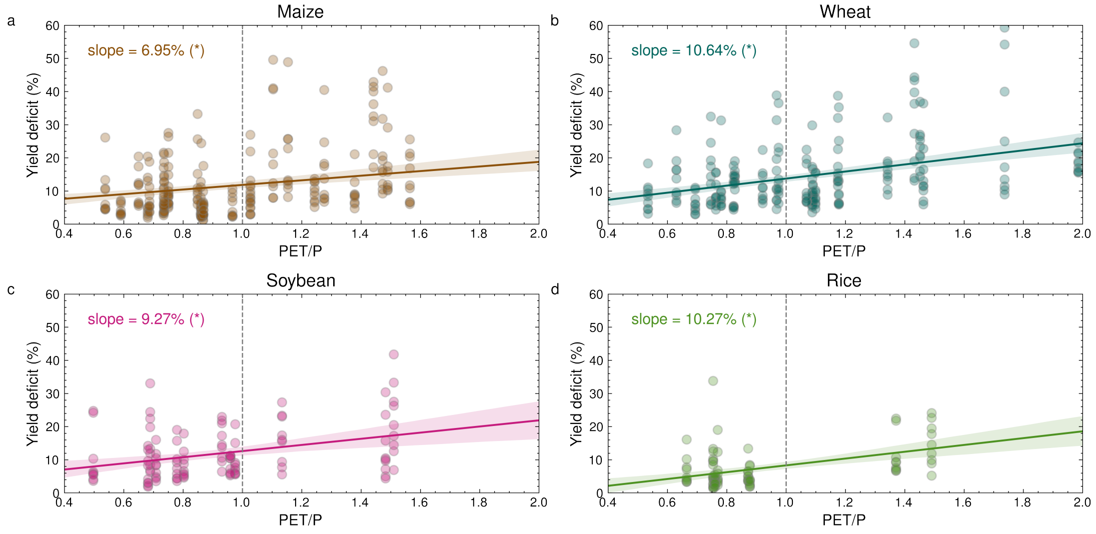

Supplementary Figure 2. Relative yield anomaly during crop failure events and climatological aridity index. The slope is estimated using an ordinary least squares (OLS) regression for maize **(a)**, wheat **(b)**, soybean **(c)** and rice **(d)**, respectively. '*' indicates statistical significance (p value < 0.05).

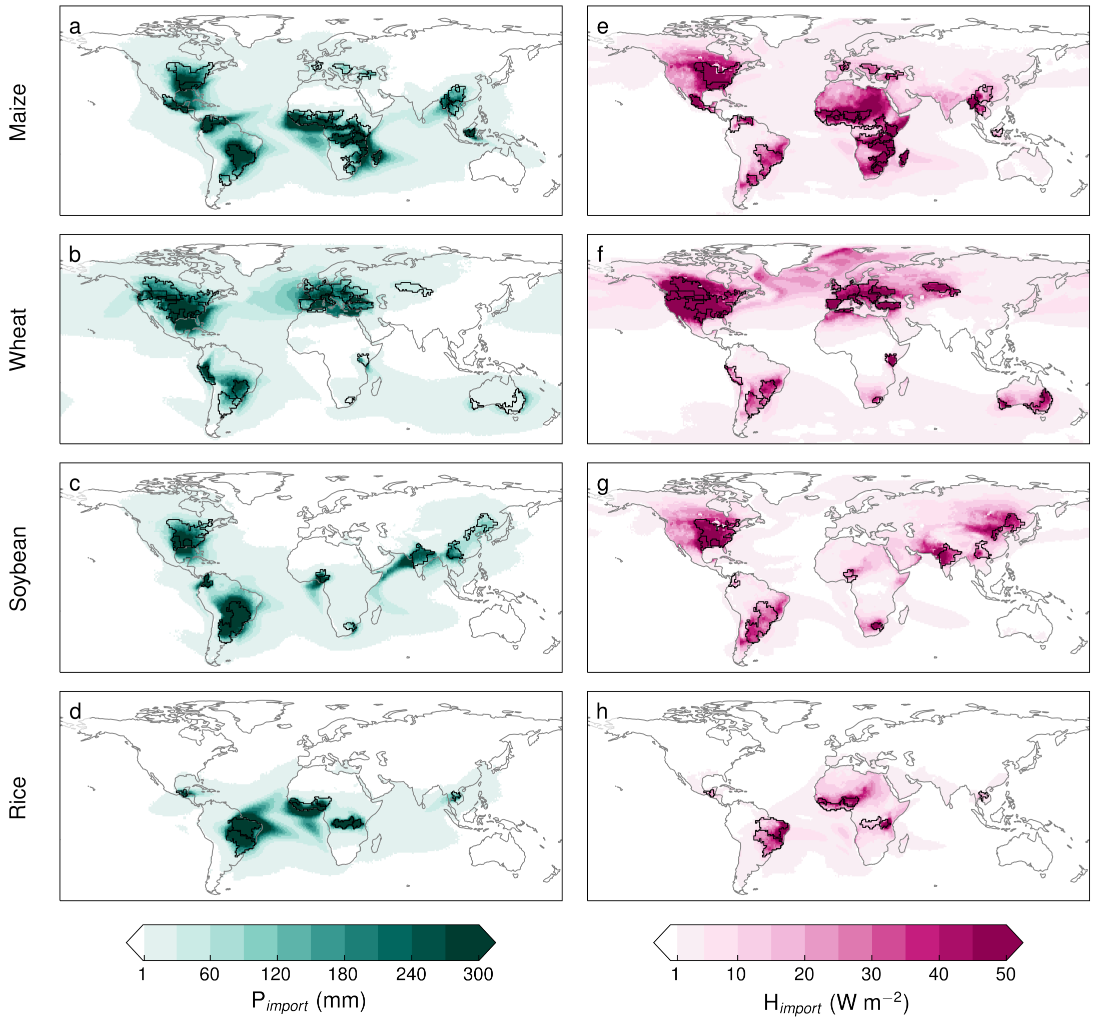

Supplementary Figure 3. Annual sources of moisture and heat imports to global rainfed breadbaskets. The sources of moisture leading to precipitation (P_{import}, mm) and heat imported into the breadbaskets (H_{import}, W m^{-2}) are shown for maize **(a,e)**, wheat **(b,f)**, soybean **(c,g)** and rice **(d,h)**, respectively.

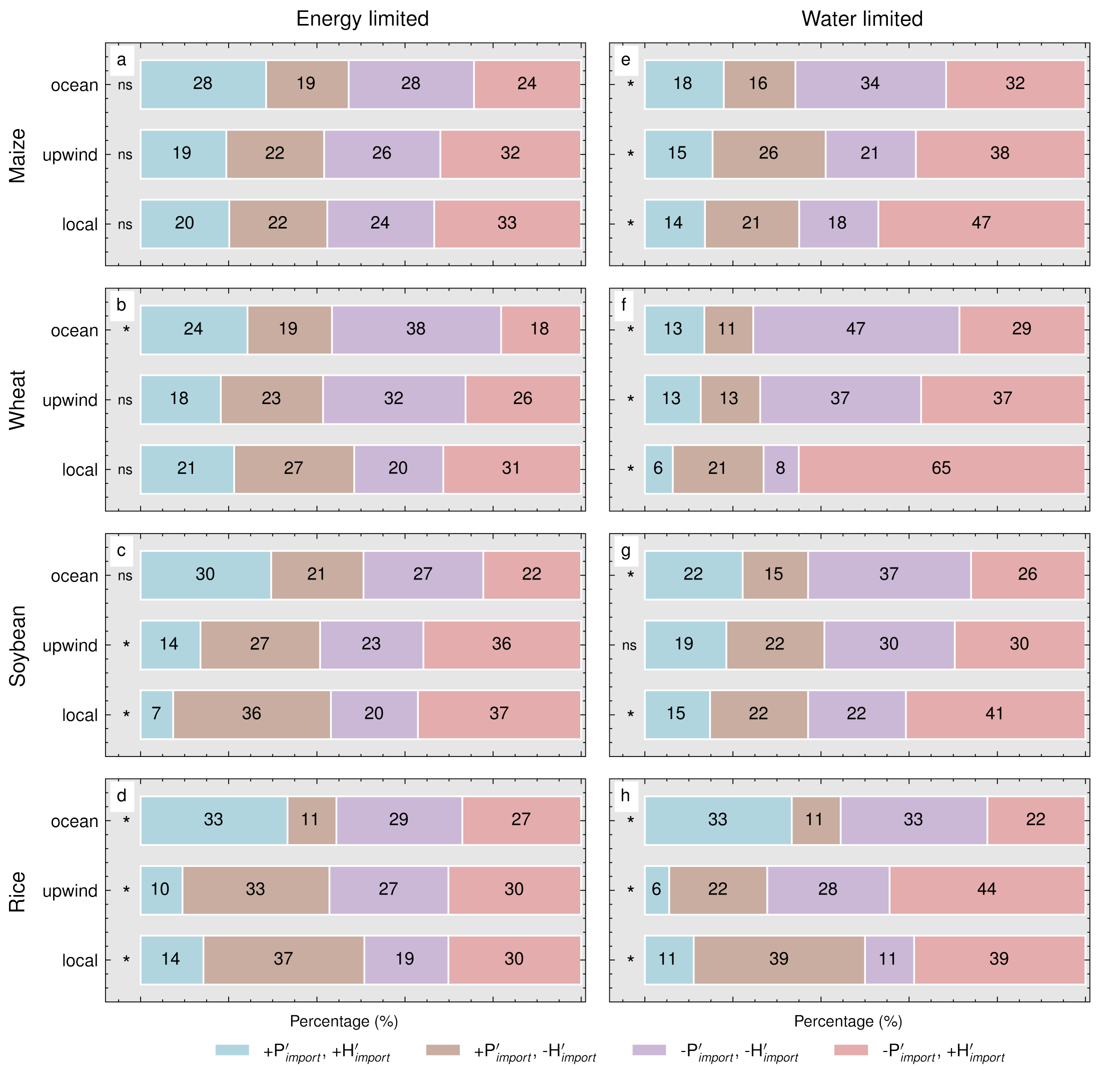

Supplementary Figure 4. Number of crop failure events in different climate conditions. Energy- **(a-d)** and water-limited **(e–h)** breadbaskets are shown for maize, wheat, soybean and rice, respectively. The 4 categories represent different combinations of positive and negative anomalies in moisture leading to precipitation (P'_{import}) and in heat (H'_{import}) imported into the breadbaskets. Differences among the 4 categories are assessed by the Chi-squared test, in which '*' indicates p value < 0.05, and 'ns' indicates non-significance.

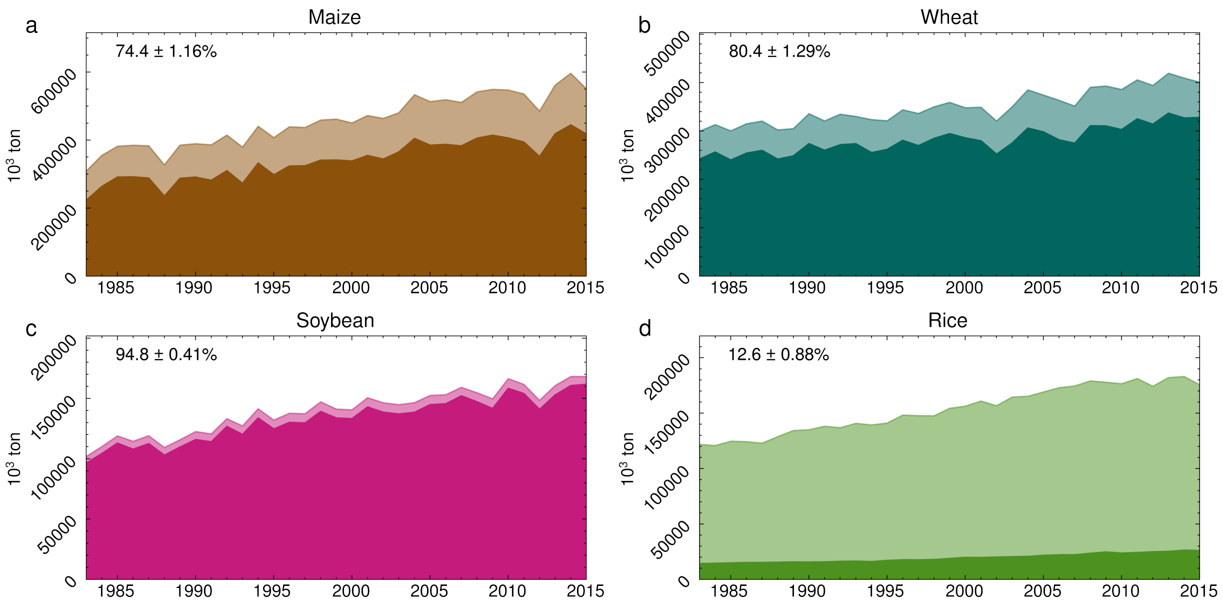

Supplementary Figure 5. Agricultural production over global rainfed breadbaskets. Annual (1983–2015) agricultural production (10^3 ton) in rainfed regions globally (light colours) and in the rainfed breadbaskets selected in this study (dark colours). Different plots indicate different crop types: **(a)** maize, **(b)** wheat, **(c)** soybean and **(d)** rice. The labels indicate the percentage ($\pm$ std) of the global agricultural production in rainfed regions that originates from the breadbaskets selected in this study.

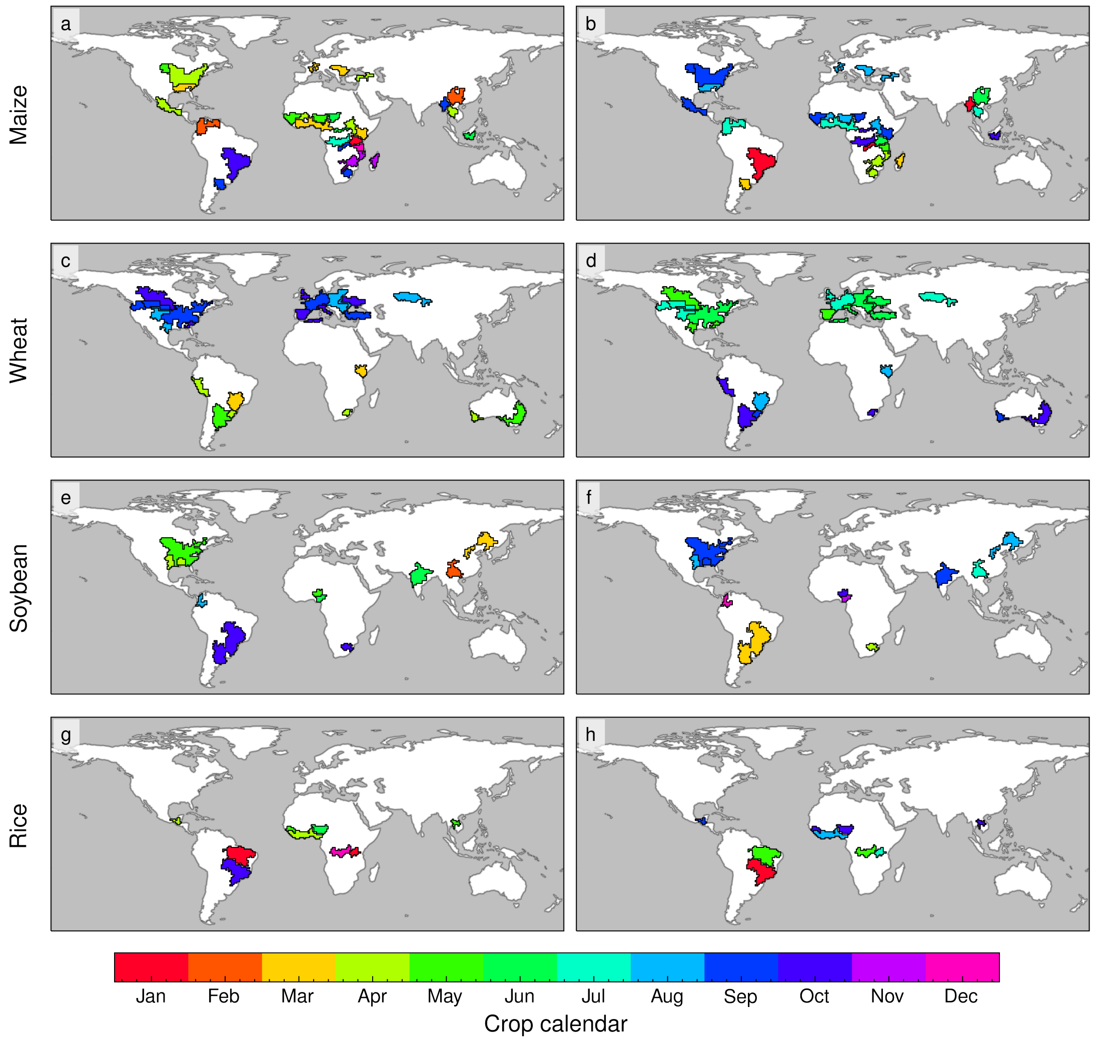

Supplementary Figure 6. Crop calendar for different rainfed breadbaskets. The sowing **(a–d)** and harvest month **(e–h)** in the year 2000 are shown for maize, wheat, soybean and rice breadbaskets, respectively.
